# Effectiveness of a virtual program for OSCE preparation during COVID-19: a descriptive and repeated cross-sectional study among nursing students

**DOI:** 10.1186/s12912-023-01396-5

**Published:** 2023-07-07

**Authors:** Rinat Avraham, Tanya Cohen, Rada Artzi-Medvedik, Nancy Hurvitz, Odeya Cohen

**Affiliations:** Department of Nursing, Recanati School for Community Health Professions, Faculty of Health Sciences, Ben-Gurion University of the Negev, POB 653, Beer-Sheva, Israel

**Keywords:** Clinical practice, COVID-19, Nursing education, Nursing students, OSCE, Virtual training

## Abstract

**Background:**

Despite the prevalence of distance learning during COVID-19, conducting clinical training for nursing students remains challenging. In compliance with social-distancing restrictions, a Zoom-based virtual OSCE preparation program for nursing students was designed, and it included clinical skills. The aims of this study were to assess nursing students’ satisfaction with a virtual program for Objective Structured Clinical Examination (OSCE) preparation, and to evaluate its learning outcomes measured by OSCE scores as compared to those of in-person preparation programs.

**Methods:**

A descriptive and repeated cross-sectional study was designed. Students’ satisfaction with the virtual program was based on a post-course survey and personal reflections. OSCE scores of graduates of the virtual program (*n* = 82) tested in 2021 were compared to those of 337 graduates of in-person programs tested in 2017–2020.

**Results:**

A post-program survey revealed that 88% of the students in 2021 were satisfied with the virtual program and felt it prepared them properly for the OSCE (26% agree and 62% strongly agree). No significant differences were found between OSCE scores following the virtual program conducted in 2021 and scores following in-person programs conducted in 2017–2020.

**Conclusions:**

This study suggests that nursing education can benefit from integrating virtual programs which incorporate clinical practices into the curricula, without harming student competency. The study results may address the problem of maintaining clinical practices in a time of limited accessibility, and in settings of low resources. It is important to expand the investigation to long-term impact of virtual training programs on nursing students’ competencies.

## Introduction

Nursing education, along with all realms of life, has undergone rapid changes during COVID-19, posing unique challenges for schools of nursing [7], enabling ongoing teaching. The relatively simple solution of switching from classroom to virtual learning made it possible to comply with the requirements for social distancing. It worked well after overcoming many initial technical problems, when students and faculty members were confronted with their low digital competence [2]. According to Gaur et al. [13], despite the social challenges of the virtual mode, such as isolation, stress, and reduced interaction with peers, distance learning gained prominence, with relatively little harm to the academic sequence of learning.

However, maintaining the clinical training of nursing students during social distancing remains a significant challenge [35], which also generated new opportunities. Carolan et al. [6] called for adopting strategies to facilitate clinical practice during COVID-19 and beyond, aimed at strengthening the resilience of the nursing educational system. For example, due to the reduction in clinical placements for students during the pandemic, many countries have pushed through legislation to switch to simulation for some of the clinical hours in the hospitals [3]. The pandemic has driven nursing education toward innovative and effective use of virtual modes in clinical training [34], with nursing schools developing virtual simulations in their efforts to maintain clinical practice [17, 27].

Objective Structured Clinical Examination (OSCE) is an accepted strategy for assessing clinical competencies of nursing students [29]. Solà-Pola [32] revealed that OSCE boosts students' confidence and helps them feel more prepared for clinical work. Following COVID-19, some schools assessed their students’ clinical competencies using virtual clinical examinations [4, 23]. Yet, it is essential to evaluate the effectiveness of these virtual programs in building students’ clinical capacity. Understanding the potential of virtual programs and examinations in preserving clinical practice of nursing students offers the opportunity to develop new educational policies and incorporate them into the nursing curriculum. Therefore, the aim of this study is to evaluate the effectiveness of such virtual program for OSCE preparation, examining students’ satisfaction and clinical outcomes.

## Background

### Objective Structured Clinical Examination (OSCE)

The OSCE is an accepted strategy for assessing clinical competencies of nursing students. Harden et al. [14] described the strategy as series of stations, in each of which the students are asked to carry out a procedure which involved demonstration various clinical competencies. OSCE benefits include greater objectivity, increased consistency of experience between students, reduced risk of examiner bias, a broader range of clinical skills tested, a high level of reliability and validity, and increased motivation for learning [20, 29]. At the same time, some limitations of the OSCE method are also mentioned in the literature, such as students’ stress, which could adversely affect their performance,complexity of the process; faculty time; high cost; difficulty in ensuring the confidentiality of the stations between different cohort of student, etc. [29]. There is a consensus among healthcare educators that it is essential to preserve OSCE during social distancing situations such as COVID-19, by adopting new performance strategies.

### OSCE during COVID-19

COVID-19 restrictions posed a major challenge for faculty to preserve OSCE and supply students with adequate preparation for the clinical examination. While some academic centers have decided to cancel the OSCE during the pandemic [30], other nursing schools reported various solutions that enable them to adjust to the COVID-19 restrictions. In several centers, where students were allowed to be physically engaged in clinical education, an in-person examination was conducted under strict limitations (e.g., [31]. Where in-person meetings were impossible, some nursing educational institutions replaced OSCE with a virtual examination [4, 11, 23].

Educators who have conducted virtual OSCEs find the Zoom application to be a suitable platform for implementation, especially the breakout-rooms feature, which creates a safe learning environment for examination and feedback [18]. Major et al. [24] reported that users considered it a promising strategy, which can be turned into telemedicine. Others call to consider the use of remote platforms for clinical training, even post‐pandemic [4, 23].

Upon reviewing results of virtual OSCEs described in the literature, students and examiners expressed overall satisfaction with the method [23], and most of the improvements needed were to overcome technical difficulties [18]. Regarding the effectiveness of the virtual exams, Arrogante et al. [4, 23] noted that no significant differences were found between scores of traditional OSCEs conducted in recent years and those of virtual OSCEs performed thus far during the COVID-19 pandemic. However, they reported difficulties in assessing clinical hands-on skills. A notable disadvantage mentioned was that students cannot practice manual skills during the test andwere required to describe their clinical skills by verbalizing to faculty, i.e., what they would perform if the treatment was in-person.

In summary, there are several gaps in the knowledge and understanding regarding strategies to maintain hands-on virtual OSCE, as well as its effectiveness in assessing clinical practices. As the OSCE usually assesses students' clinical skills proficiency, it is essential to find ways that will assist faculty to evaluate actual clinical performance via virtual examination. Reports on virtual OSCEs also highlight the importance of the preparation phase for both students and staff [24].

### The OSCE preparation virtual program

The OSCE format used in the Department of Nursing at Ben-Gurion University of the Negev, is a “Multi station OSCE” [29], and is used as a final exam in the "Critical Clinical Thinking and the Nursing Process" course, part of the second-year baccalaureate curriculum. There are usually six OSCE stations, each taking 10–12 min, with a 2-min break between them. The clinical skills evaluated are taking medical history, nursing assessment for a holistic approach, clinical-manual skills, patient-education skills, critical thinking, and clinical decision-making principles. All stations are directly observed by faculty members of the Department of Nursing, who are also part of the teaching team. Evaluation during the exam is based on binary structured checklists and a global rating scale [20]. Every year, as a part of the students’ preparation for the OSCE, an in-person preparation program with two clinical stations is conducted. This program aims to demonstrate an OSCE station and to enable students to experience this test.

The OSCE preparation virtual program was designed for second-year baccalaureate nursing students, based on the INACSL Standards of Best Practice: Simulation^SM^ [15]. The program was conducted on January 18, 2021, during a total lockdown imposed by the pandemic. Due to the lockdown restrictions, and in accordance with the university lockdown rules, students were not allowed to come to the campus for the preparation program, but were required to take the final OSCE in-person. To meet these restrictions, we designed a similar virtual OSCE preparation program via Zoom (instead of the traditional in-person preparation program), which was directed to prepare students for the in-person OSCE.

Program design and procedure:

- Stage 1—Stations development

Two scenario-based OSCE stations were developed and designed to be as similar as possible to the real (hands-on) exam. Appropriate checklists were adjusted for enabling the evaluation of students' performance via Zoom. Table 1 presents the scenario details of each station.

- Stage 2 – Equipment arrangement

To enable the trial of clinical skills, an equipment kit had been organized and delivered to the students a week before the program began. The kit included syringes, needles, tubes, a fluid bag, IV connection set, micro enema, water for injection (WFI) ampoules, and disposable protective equipment (e.g., gloves and apron). The students were instructed to keep the equipment kit closed until the program began. For practice needs, students were instructed to prepare a disposable drinking bottle with a narrow mouth, which was used to represent the patient.

- Stage 3 – Faculty and students’ instructions

A few days before the program began, preparatory Zoom meetings were held to inform students and teachers of the program schedules, goals, and methods, and instruct them on how they should prepare. Students and teachers received the schedule, and Zoom links for each of the stations were sent following the preparatory meeting.

- Stage 4 – The virtual program

**Fig. 1:**
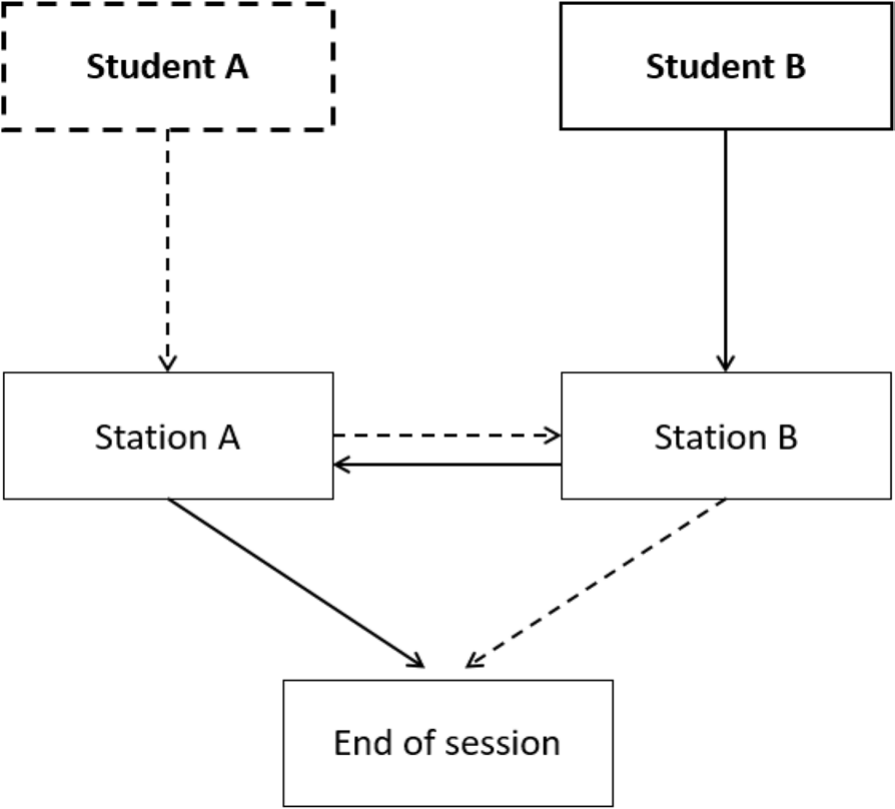
Flow chart of the program for a single student

Each student practiced at two virtual OSCE stations via Zoom. Each station session lasted 10 min. The student entered the first Zoom meeting, where a teacher was waiting for her/him. At the beginning of each session, the scenario was presented to the student. During the time at the station, the student was required to collect relevant clinical information from the patient’s medical records, assess the patient, decide on the appropriate care, present the patient to the doctor and ask for a medical order, perform the medical order (a specific clinical procedure), and evaluate the intervention results. The teacher shared all the required materials on the screen. The student practiced manual skills on the disposable drinking bottle using the equipment kit. After 10 min, the teacher announced the end of the session and gave the student a short debriefing. Then, the student left the first station Zoom meeting and proceeded to the second station, in which another case was presented. Figure 1 illustrates a student's path through the sessions.

- Stage 5 – Evaluation

Using a structured checklist, a teacher evaluated the student's performance at each station. As this was a preparatory program for OSCE, students were given a 10-min personal debriefing at the end of each station by the teacher who observed their performance. The debriefing reflected what went well during the session, and what skills require additional training. Following the virtual sessions, students' satisfaction was evaluated using quantitative and qualitative methods.

**Table 1:** OSCE preparation virtual program stations characteristics

Characteristics	Station 1	Station 2
Patient and setting	45-year-old woman who arrived in the ER	38-year = old postpartum hospitalized woman
Medical condition	Chronic anemia with orally treatment of iron supplements	s/p Spontaneous vaginal labor using epidural block
Current situation	Abdominal pain, flatulence, and constipation	Urinary catheter "bothers her"
Competencies	History taking, patient assessment and clinical skill (micro enema)	History taking, patient assessment and clinical skill (urinary catheter exertion)

The study leans on Kirkpatrick and Kirkpatrick's [22] four-level evaluation model for educational learning outcomes. This model is commonly used for evaluating the effectiveness of health professionals’ educational programs, and especially useful in helping evaluators identify learner outcomes [12]. In this study, we assessed the virtual program's effectiveness through the first two levels of Kirkpatrick and Kirkpatrick's model [22]: Level – evaluation of participants’ reaction (in terms of participants' satisfaction to the educational program,Level 2 – evaluation of the learning generated by the program, demonstrating the influence of the educational program on participants’ knowledge, attitudes, and/or skills.

The study aims were (1) to assess nursing students’ satisfaction with the virtual OSCE preparation program, and (2) to explore the learning outcomes of the virtual program by comparing the OSCE scores obtained following virtual preparation to those obtained following in-person preparation. According to this theoretical model we hypothesized that:

- *Hypothesis 1.* Participants will be satisfied with the virtual OSCE preparation program.
- *Hypothesis 2.* The learning outcomes measured by the final OSCE scores following the virtual preparation program will be similar to those achieved following an in-person preparation program.

## Methods

### Design

The study used a descriptive and repeated cross-sectional design.

### Participants

Participants were second-year baccalaureate nursing students in the Department of Nursing at Ben-Gurion University of the Negev between 2017 and 2021. Inclusion criteria were participation in the virtual OSCE preparation program conducted in 2021, or in the in-person OSCE preparation in 2017–2020.

### Data collection

Participants’ satisfaction with the virtual preparation program was measured among students who had participated in the program (*n* = 82), using a single item (as suggested by [5] from the general evaluation survey for the course: "I am satisfied with the virtual OSCE preparation program.” Answers were rated on a 4-point Likert scale (1* – strongly disagree* 2* – disagree,* 3* – agree,* 4* – strongly agree*). Additionally, an open-ended question gave participants the opportunity to express their subjective, personal experience with the program.

In order to evaluate learning outcomes, we compared the OSCE scores following the virtual program in 2021 to those achieved following in-person preparation in 2017–2020 (*N* = 419). The score of an OSCE station is based on binary structured checklists, including about 30 expected clinical behaviors to be performed at each station, followed by a global rating scale, assessing the quality of student’s performance [20]. Calculating an OSCE station score is based on combining the results of the binary structured checklist, 90%; and the global-rating scale, 10%. The final OSCE score is calculated as the mean score of all OSCE stations. We compared the final OSCE scores means, and the mean scores of four selected OSCE stations that were found to be similar all over the study duration: patient education, IV administration, respiratory assessment, and pain management. Content validity of the checklists was measured by at least two faculty members, who are experts in fundamental of nursing education. Demographic information for each year was retrieved from the administrative unit at the Department of Nursing.

### Ethical considerations

The study was approved by the Head of the Nursing Department, and by the Institutional Review Board at the Faculty of Health Sciences of Ben-Gurion University of the Negev (request #35–2021).

### Data analysis

Analyses were conducted using IBM Statistic SPSS software version 26. Descriptive statistics were calculated to explore participants' profiles and study scores. Chi-square tests examined differences among students. A multivariate analysis of variance (MANOVA) with Scheffe post-hoc analysis was performed to compare the effect of the pre-pandemic and during pandemic final OSCE scores. Analysis of the open-ended question was used to strengthen descriptive statistics regarding participants’ satisfaction.

## Results

### Participants

The study was conducted on 419 s-year baccalaureate nursing students between 2017 and 2021. Table 2 presents the age mean and standard deviation, and the gender frequency of participants over the study period. No significant differences in students' characteristics were found over the years.

**Table 2:** Demographic characteristics of study's participants (*N* = 419)

Characteristic	2017 (*n* = 81)	2018 (*n* = 92)	2019 (*n* = 77)	2020 (*n* = 87)	2021 (*n* = 82)
Women	88%	88%	84%	93%	94%
Men	12%	12%	16%	7%	6%
Mean age	25.1 (SD = 2.34)	25.9 (SD = 1.92)	25.36 (SD = 3.64)	25.07 (SD = 2.04)	24.9 (SD = 1.7)

### Main results

Evaluation of students’ satisfaction from the virtual preparation program revealed that 79 (96.3%) students ranked their satisfaction with the virtual program. Most of them were strongly satisfied or satisfied with the virtual program (*n* = 49 (62%) and *n* = 21 (26.5%), respectively), while only nine students (11.5%) were not satisfied. Students' reflections in the open question were generally positive, and expressed high satisfaction from the program, for example:

The practice was just great! Very good teaching and preparation, and I came out with a great feeling.… The attention to small details, the preparations, the equipment, the professional guidance, the educational level. I feel much better prepared for the exam. There really are no words to say except a big thank you!

The kits that were delivered, and the training that was performed (even though it was via Zoom)… it was evident that a lot of thinking and a lot of effort was invested despite all the COVID-19 restrictions.

I wanted to say thank you for your creative thinking and willingness to help and find creative solutions during this complex period.

**Fig. 2:**
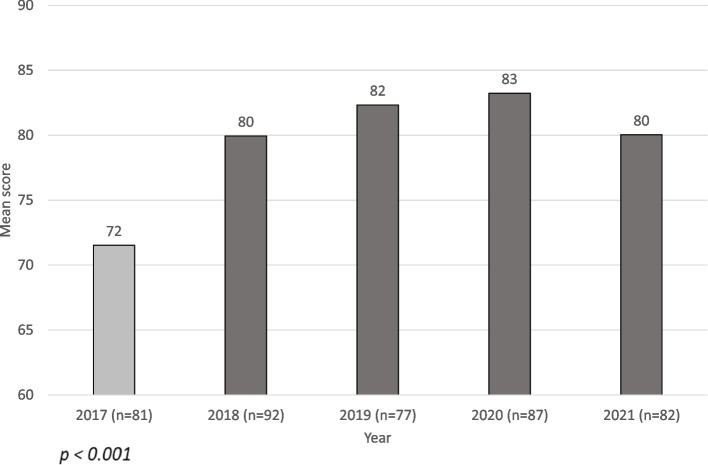
OSCE final grades over the years. Note: Different colored bars indicate a significant difference based on MANOVA

Evaluation of the learning outcomes of the virtual program as reflected in the OSCE scores revealed that as hypothesized, no significant differences were found in the OSCE scores following the virtual preparation program conducted in 2021 and the scores following the traditional in-person programs conducted in 2017–2020. Figure 2 presents the final OSCE scores over the years. As depicted, no differences were found between scores in 2018–2021. The only significant difference was between the score of 2017 and the scores achieved in all other years of the study (*F* (4, 414) = 36.666, *p* < 0.001).

**Fig. 3:**
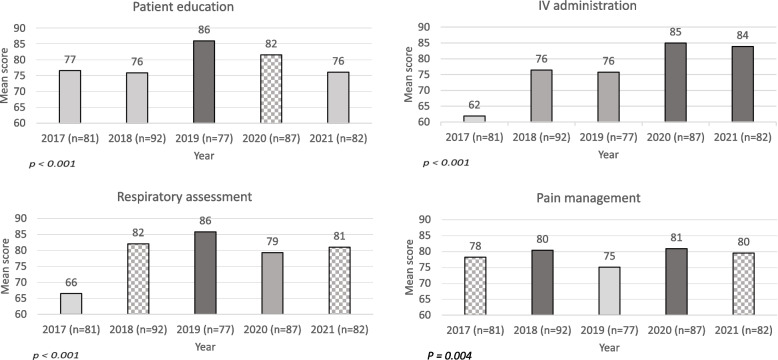
Mean scores of selected OSCE stations over the study years. Note: Different colored bars indicate a significant difference based on MANOVA. A bar with patterns presents a non-significant difference between close values

Exploring the scores of the four selected stations, revealed un-specific trends. The results show a significant effect of the year of study, V = 0.825, F(20, 1652) = 21.467, *p* < 0.001.. However, post-hoc analysis revealed that these differences did not rise specifically following the virtual preparation program. Indeed, the 2021 scores were found to be similar to other scores during the study. Figure 3 presents the mean score prevalence of the four stations over the years. For example, in the IV administration station, students achieved the highest scores in 2021 similarly to 2020 (*F* (4, 414) = 64.679, *p* < 0.001), while in the patient education station, the 2021 scores showed no significant differences from those of 2020, 2018, and 2017. The significant difference in this station stems from the high score in 2019 (*F* (4, 414) = 8.398, *p* < 0.001). Detailed OSCE scores with MANOVA results are presented in Table 3.

**Table 3:** OSCE parameters with MANOVA results

					Scheffe		
					subset for alpha = 0.05		
Variable	Year	Mean	Std. Deviation	MANOVA	1	2	3
Final grade	2017	72	0.605	F (4, 414) = 36.666, *p* < 0.001	72		
	2018	80	1.006			80	
	2019	82	0.483			82	
	2020	83	0.642			83	
	2021	80	0.826			80	
Patient education	2017	77	1.558	F (4, 414) = 8.398, *p* < 0.001	77		
	2018	76	1.635		76		
	2019	86	1.06			86	
	2020	82	1.338		82	82	
	2021	76	1.769		76		
IV administration	2017	62	0.922	F (4, 414) = 64.679, *p* < 0.001	62		
	2018	76	1.35			76	
	2019	76	1.34			76	
	2020	85	0.968				85
	2021	84	0.997				84
Respiratory assessment	2017	66	0.637	F (4, 414) = 35.050, *p* < 0.001	66		
	2018	82	1.349			82	82
	2019	86	1.116				86
	2020	79	1.296			79	
	2021	81	1.451			81	81
Pain management	2017	78	1.176	F (4, 414) = 3.956, p = 0.004	78	78	
	2018	80	1.234			80	
	2019	75	1.059		75		
	2020	81	1.211			81	
	2021	80	1.026		80	80	

## Discussion

This study designed in accordance with Kirkpatrick and Kirkpatrick's [22] evaluation model for educational learning outcomes. We assessed the virtual program's effectiveness through the first two levels of model: level 1 – evaluation of participants’ reaction (in terms of participants' satisfaction) to the virtual program aimed to prepare them for the OSCE,and level 2 – evaluation of the learning outcomes by comparing their OSCE scores to those achieved by students who had undergone the traditional in-person program. Our results show that following the virtual program conducted during the pandemic, students’ satisfaction was high, and they felt that the faculty responded to their needs. Additionally, it was found that OSCE scores following the virtual preparation program were similar to those of previous years, indicating that the virtual program prepared them as well as the traditional in-person programs conducted prior the pandemic.

The use of the Zoom in health education was expanded during the COVID-19 pandemic to a wide range of locations, including Low- and Middle-Income Countries (LMIC; [10]. Weine et al. [36] argue that using remote education during the COVID-19 among LMICs, overcome not only the limitations of existing inequitable models of engagement and global health education but also faces future challenges by providing the needed support to LMIC partners to participate more equally. During the last years, the possibility to use personal training models for individual clinical training is increasing. However, this study used common and cheap means as a disposable drinking bottle for demonstrating the patient, enabling it to be implemented in low resources locations, or in situations when the preparation does not require the use of dedicated and unique means.

### Students’ satisfaction

The assessment of students’ satisfaction is crucial in integrating new strategies into the nursing curriculum. Trainees’ satisfaction is the basic stage of program-evaluation models [22], enabling feedback for the program developers. Furthermore, Doménech-Betoret et al. [9] highlighted that academic satisfaction and performance are positively associated with student’s self-efficacy, which is essential for academic success. Focusing on the learning methodology, Kim and Park [21] showed that nursing students’ satisfaction with distance e-learning mediated the influence of learning flow and learning outcomes (defined as the final goal when evaluating education). Students who are satisfied with the learning program may be better engaged in learning and show greater involvement [19]. Creating new educational programs during the pandemic in a short time is challenging, and evaluating students’ satisfaction is warranted, especially if schools are considering using virtual programs beyond the pandemic.

Evaluating students’ satisfaction is also essential for the faculty, as greater satisfaction may be associated with higher trust, thus leading to better relationship between students and faculty [26]. Furthermore, while students’ negative feedback on a new educational program generated negative feelings among faculty (e.g., disappointment and frustration), students’ positive feedback that reflects great satisfaction may generate positive feelings thus enhancing faculty motivation and commitment to create more innovative programs that meet students’ needs.

### Learning outcomes

In our nursing department, the “Multi-station OSCE” method [29] has been used since 2017. The results of this study provide a perspective for the entire study period, enabling an analysis of learning outcomes reflected by OSCE performance. The present study is consistent with other studies conducted during COVID-19 [4, 17, 23, 27], emphasizing the challenges in maintaining clinical practice and evaluation – the core of nursing education [25]. The described virtual preparation program was unique in that it succeeded in preparing students for an in-person OSCE. In addition, our findings are corresponding with other studies that found no significance differences between virtual and in person the nursing education (e.g. [37].

Thus, we suggest that virtual clinical learning may be integrated into traditional programs, without harming students’ competencies. Although the virtual preparation program was developed specifically for the COVID-19 lockdown, following this study, similar programs could be designed and be used in the nursing curricula post-pandemic both to address other situations of limited access to clinical practice, and to expand students’ possibilities to acquire and improve competencies during their studies.

Although this virtual preparation program was developed for solving the problems imposed by the lockdowns during the pandemic, virtual programs may have broad implications for the nursing and medical educational system: First, virtual teaching has high availability, time, and location flexibility [8], with an increased potential of strengthening students’ clinical competencies [38]. Second, considering the shortage of clinical training resources [33], virtual programs may partially replace clinical rotation’s hour requirements. Hence, further evaluation of the effectiveness of this replacement is needed. Third, these alternative teaching methods enable the continuity of clinical teaching and practicing when the primary mode is not available (for any reason), thus possibly strengthening the flexibility and resilience of the nursing educational system [30]. Fourth, virtual clinical practices enable students to acquire new skills of using technology in the nursing arena. As telehealth became highly common during the pandemic and is expected to thrive in the future [16], additional learning outcome of the virtual training may be that students learn how to deliver healthcare on a virtual platform effectively (e.g., patient-nurse communication, patient teaching and consultation). Following former studies, advancing the integration of clinical simulation requires supportive policy, and could not be achieved by individual efforts [1]. Park and Yu [28] highlighted that formal standards are needed to improve the overall flow of nursing education, including exposure to various methods' applications, training the trainers and the importance of trained nursing faculty.

### Limitations

The results presented in this study have several limitations. First, this study was conducted in one nursing educational center; further studies may expand the study sampling. In addition, sample size was not determined since sampling was based on a retrospective data. Second, OSCE scores may be influenced by students and faculty characteristics that were not considered in this study. However, the duration of this study decreases this possible bias. Third, this study measured the general satisfaction via a single item used to assess students’ satisfaction as suggested by Assunção & Pimenta [5]. Further studies may focus on other satisfaction domains.

## Conclusions

This study suggests that nursing education can benefit from integrating virtual programs for clinical practices, without harming student competency. These findings may have implications for the nursing educational system, by supporting policies of virtual program implementation as a new and promising training method in the nursing curricula. Further research exploring and assessing the effectiveness of virtual programs on students’ competencies in the clinical setting is warranted.

## Data Availability

The datasets used and analyzed during the current study are available from the corresponding author on reasonable request.

## Abbreviations

MANOVA: Multiple analysis of variance
OSCE: Objective Structured Clinical Examination
